# Development of a computer-assisted instructional package for life skills to prevent risky sexual behaviors in early adolescents, Bangkok, Thailand

**DOI:** 10.12688/f1000research.27773.2

**Published:** 2022-01-11

**Authors:** Wanida Neranon, Ladaporn Thongsong

**Affiliations:** 1 Department of Pediatric Nursing, Kuakarun Faculty of Nursing, Navamindradhiraj University, Bangkok, 10300, Thailand

**Keywords:** Prevent Risky Sexual Behavior, Early Adolescents, Activity Package, Computer Assisted Instruction, Life Skills, Development

## Abstract

**Background: **The purpose of the present study was to examine the effects of a newly developed computer-assisted instructional package for life skills (CAIFLS) specifically designed to raise awareness of risky sexual behaviors among Thai early adolescents in Bangkok.

**Methods**:  The research process included two phases: (1) the development and (2) the use and evaluation of the newly developed CAIFLS package. First, 5 teachers and 5 Grade 7 students of a Bangkok school were interviewed to collect information needed for the development of CAIFLS. The second phase was to implement learning activities through CAIFLS with a total of 87 Bangkok school students, consisting of 44 students for the experimental group who received CAIFLS for 4 sessions, and 43 students for the control group who received routine class lecture. CAIFLS instructions, lesson plans and worksheets were designed as the experiment methods. Then questionnaires of life skills assessments and student satisfaction were used to investigate the effects and the student satisfaction of CAIFLS.

**Results**: The findings revealed that the efficiency values of the CAIFLS package were 80.2/82.5, higher than the set criteria of 80/80. Mean scores on life skills for the experimental group significant increased (p < .05), which was higher than the control group. The students also showed their satisfaction of CAIFLS at a high level (M = 4.20, S.D. = 0.29)

**Conclusions**: CAIFLS can be used as an effective learning tool to enhance life skills to prevent risky sexual behaviors among Thai early adolescents.

## Introduction

Sexual values and behaviors in Thai society are determined through the learning system cultivated from society, education and family. In the past, sex education and premarital sex were considered inappropriate behaviors. Schools, therefore, did not teach sex education as it was supposed to be taught because it was believed teaching sex education in a straightforward manner could lead to sexual activities. As a result, the core principle of sex education often emphasized a ban on premarital sex ( Achavanichakul, 2011). In Thai society, there was the value that women had to be reserved and refrained themselves from any sexual activities that indicated women’s worth. Therefore, Thai women had been disciplined to be careful and control their sexual behavior since they were children in order to prevent premarital sex and refrain themselves from expressing or being open to sex in the public ( Simuangngam, 2011). However, nowadays sexual values and behavior are bound to change. According to the National Statistical Office Survey on Conditions of Society and Culture 2011, there was tremendously increasing tendency in the proportion of the sexual behaviors among early adolescents since school age children as compared to the past ( National Statistical Office, 2012). The risky sexual behaviors of Thai early adolescents revealed their sexual initiation at a young age that reflected the riskier behaviors among early adolescents.

High-risk sexual behaviors, especially in early adolescents, are one of the primary concerns in many countries. In Thailand, it was reported that the average age of early adolescents’ first sexual intercourse was 12 years ( Bureau of Reproductive Health, 2017). Statistics in 2014 showed that female adolescents aged 15-19 gave birth at the rate of 47.9 cases compared to 1,000 among the entire population at the same age ( Bureau of Reproductive Health, 2017). The tendency of child pregnancy seems higher, which leads to physical, mental, economic and social problems, e.g. abortion, sexual transmitted diseases, divorce and lack of experience in taking care of children ( Unite of children, 2015). In the 2015 academic year, of 8,814 cases of student dropout, 4,019 cases were lower secondary school students ( Chuaykaew et al., 2018; Ministry of Education, 2016). According to a survey conducted by the Bureau of Reproductive Health, Department of Health, Ministry of Public Health, 2011, consistent with ( Thiammok & Saranrittichai, 2017) related to sexual risk behaviors among early adolescents in Bangkok, early adolescents admitted to being involved in the following sexual behaviors: 47 percent of them held hands. 33 percent cuddled. 29 percent had sex with lovers or male/female friends. 61 percent of who had sex had more than 1 sex partner, whereas more than 50 percent were indifferent to the importance of the use of condoms. The aforementioned behaviors can lead to poorer academic performance, adolescent pregnancy, sexually transmitted diseases, and AIDS. In addition, it was found that the childbirth rate of Bangkok teenagers aged 15 to 19 years old was at 43.30 per 1,000 female adolescents in 2013-2015 ( Bureau of Reproductive Health, 2017). This, together with the problems mentioned earlier, was likely attributed to Thai early adolescents’ lack of sexual health knowledge (e.g., sexual relationship, high-risk sexual behavior, birth control) despite the fact that sex education is mandated and promoted in schools ( Mahanta et al., 2016).

Preventing early adolescents from having premature sex should be started when they enter reproductive age, which is between the ages of 12 and 13 (Grade 7) to prevent sexual behavior problems in the future. Based on the analysis of a thesis of public health nursing in children and early adolescent in schools, life skills can influence behavioral changes ( Widman L, et al., 2016). Life skills development is crucial for early adolescents, for example, to think critically, solve problems appropriately and communicate effectively, especially in prevention of sexual behaviors ( Paknoi, 2017; Prabmeechai & Rueangworaboon, 2017). However, from previous studies it has been seen that Thai early adolescents have insufficient life skills in problem solving; resulting in inability for self-adjustment and making developmental changes ( Bunnag et al., 2013; Butcharoen et al., 2012). The practical use of life skills is significant for their daily life, and adolescents should maintain appropriate skills to protect themselves from risky sexual behaviors. The World Health Organization has defined ‘Life Skills’ as “abilities for adaptive and positive behaviors that enable individuals to deal effectively with the demands and challenges of everyday life” ( World Health Organization, 1994).

Previous research has studied the development of student training courses to prevent sexually risky behaviors in a secondary school in Bangkok and found that students needed to learn about gender-related life skills in four areas, namely relationship building skills, communication-denial skills, self-esteemed skills, and self-awareness skills. Relationship building skills are the abilities to build good relationships with each other, to understand the differences between three stages of relationship (Stage 1 - Friendship, Stage 2 - Love, and Stage 3 - Sexual relationship) and to maintain friendship or non-sexual relationships. Communication-denial skills are the ability to communicate or express one’s feelings of thought appropriately with culture and circumstance through, for example, opinions, negotiations, or denials. Self-esteem is the ability to control and value one’s self by not having risky sexual behaviors, including dating, hanging out or being alone with friends of the opposite gender in private places, reading or watching media that have sexual content, kissing, touching, or drinking alcoholic beverages before having sex. The fourth area of self-awareness is the ability to observe, know and understand one’s self (e.g. strengths and weaknesses, needs) as well as to be aware of inappropriate school-age sexual behaviors that can have an impact on their body, mind and society. These four life skill aspects in relation to sexual behaviors are also found in literature in that if students lack relationship building skills, they will not be able to distinguish an emerging relationship, whether it is friendship or love, which could destroy good relationships between friends ( Sirited P, 2018: Thiammok & Saranrittichai, 2017: Wongjunthong & Rachawijit, 2010). Also, if they do not possess communication-denial skills, they might use inappropriate denial strategies (e.g., involving aggression) when disagreeing on opinions or denying requests in relation to high-risk sexual behaviors from others ( Kirdin S et al., 2019: Olanratmanee B, 2018). Students who lack self-esteem skills will not be able to control or value themselves. There is a trend among early adolescents that having a partner in school age or having multiple partners is currently acceptable. Inappropriate sexual behaviors between men and women, e.g. touching body parts, going clubbing, wearing seductive dresses, drinking alcohol or using illegal drugs, has led to an increasing sexual intercourse rate ( Butcharoen et al., 2012: Kylene G. et al., 2012). In addition, if the students lack self-awareness, they will not be able to understand themselves or to identify their strengths and weaknesses ( Kosalavit T et al., 2017). Therefore, enhancing life skills among early adolescents is very important in order to help them protect themselves from risky sexual behaviors they might be facing in society. Teaching life skills needs the participation of early adolescents to reflect their thoughts and feelings from their previous experiences, build interactions, and create new knowledge together.

A review of life skills in preventing sexual risky behaviors of early adolescents and interviews with students found that preventing sexual risky behaviors focuses on sex education, positive attitudes towards sex, and life skills teaching through lectures, group discussions, role-playing, with the use of various types of media (e.g. brochures, videos, short films, demonstrations) ( Tungsaengsakul S et al., 2017: Kosalavit Tet al., 2017). To the best of our knowledge, computer-assisted instructions (CAI) has not been adopted to promote life skills in preventing sexually risky behaviors, but has been used to promote sex education in early adolescents in Thailand ( Wongjunthomg K & Rachawijit J, 2010: Eamratsameekool W, 2008). However, currently early adolescents, who have been raised and surrounded by new technologies and various media, have unique learning styles, need freedom and want to learn new things through innovative and interactive platforms. . It is evident in the literature that young adolescents can learn things quickly and easily from innovative media around them (Bertrand, 2006: Sangchart E & Duangsong R.,2016). Thus, media used for their learning should be suitably designed to help stimulate their interest in learning. CAI is one learning tool with features for presenting multi-media content on a computer that can promote student learning and also life skills to prevent sexually risky behaviors among early adolescents ( Alessi S.M. & Trollip S.R., 1985; Bualoy et al., 2014; Sasinun et al., 2013). It can help students to learn difficult content more easily in a short time, which responds to students' needs and self-learning ( Kanokthavomtham A & Lalognum N, 2017; Krungkraipetch, 2019). CAI is also a virtual-reality teaching medium that plays a role in education by using computers to help create learner-centered lessons ( Ratta S. et al., 2015). It promotes self-learning through new experiences from characters, animations, colors, and sounds that help attract students to interact with the lessons and increase learning motivation and understanding ( Wongjunthomg K & Rachawijit J, 2010: Chaisatsampun W & Jittrapirom A, 2017).

Thus, the present study is aimed at developing and studying the results of a series of computer-assisted lessons to promote life skills in preventing sexual risky behaviors in early adolescence. It is hoped that the newly developed CAI lessons can reduce school-age sexual intercourse, teenage pregnancy, or sexually transmitted diseases.

## Methods

### Ethical considerations

This Research and Development Project was approved by the Institutional Review Board, Kuakarun Faculty of Nursing, Navamindradhiraj University (approval number KFN-IRB2018-19 and KFN 22/2019). Information about the study was explained to the students; objectives, data collection steps, timing of research study, and benefits of participation in the research project were given to the students, their guardians and teachers prior to conducting the study. Written informed consent to participate in the study was signed and obtained from the students and their legal guardians. They were paid for participation in this research project, as approved by the Research Fund Board of the University. Upon completing the interview, each participant received 300-baht cash incentive for their participation.

### Study design

The research project was divided into two phases; phase 1 developed the computer-assisted instructional package for life skills (herein ‘the CAIFLS’). In phase 1, the CAIFLS was validated using pilot testing. In phase 2, the efficacy of CAIFLS was tested in a group of secondary school students using a questionnaire. In phase 2, student satisfaction with CAIFLS was also evaluated.

### Phase 1

*Development of CAIFLS package*

To understand life skills (LS) in preventing sexual risky behaviors in Thai early adolescences, the CAIFLS and a questionnaire were systematically developed by the researchers. First, background information was synthesized by reviewing textbooks, research studies, articles. After the researchers reviewed and synthesized the theory of LS and related research, a group of participants in a school in Bangkok were sampled to participate in interviews for a situation analysis of current risky sexual behaviors in the school.

The participants in the interviews were purposively sampled from two target groups of participants, i.e. students and teachers. Five students were selected based on the criterion of differences in their academic achievements (GPA) (1 high-, 2 moderate-, and 2 low-GPA students) and gender (3 females and 2 males). The second group consisted of 5 teachers who were responsible for guidance, advisory teacher, Health-Physical Health Education and administration. Two sessions of group interviews with teachers and students were conducted to gather information about current high-risk sexual behaviors in school students ( *Extended data* ( Neranon & Thongsong, 2021b)). Then the researchers analyzed the collected data from the group interviews and the contents, including life skills needed to prevent or reduce the sexual risk behaviors in early adolescents, guidelines for promoting life skills in preventing early adolescent sexual risk behaviors, and the characteristics of preferred teaching materials. After the data analysis, the researchers developed a series of computer-assisted lesson activities to promote life skills in preventing sexually risky behaviors for early adolescents in four main aspects: 1) relationship building skills; 2) communication–denial skills; 3) self-esteem skills; and 4) self-awareness skills.

CAI lesson series entitled ‘Adolescents and life skills on sexual self-prevention’

The CAI was divided into three steps ( Alessi S. M & Trollip S.R., 1985): 1) planning step: comprised of define the scope, cost the project, produce a planning document; 2) design step: comprised of develop initial content ideas, conduct task and concept analyses, preliminary program description, prepare a prototype, create flowcharts, storyboards and prepare scripts; 3) development step: write program code, create the graphics, produce audio and video, test and make revisions.

The final version was developed in the form of Adobe Flash Player. It features two-choice pre- and post-tests to measure life skills to prevent sexual risky behaviors in early adolescents. Once the students complete the tests, they can see the scores they have achieved. For the post-tests, the students can see the correct answers of all items. Students’ pre- and post-test scores are recorded in the program. The program was created and designed using still images and animations with vivid colors, voiceover, and subtitles. Four lessons were developed based on situations that students encounter in everyday life. The series of CAI included: 1) relationship building skills; 2) communication–denial skills; 3) self-esteem skills; and 4) self-awareness skills.

The stories are based on situations students encountered in their daily lives. Each lesson contains the following objectives:

Lesson 1: Relationship building Skills. This story is based on a simulation. Its objective is to create the ability to build good relationships between them and understand 3 stages of a relationship: The 1st stage is friendship; the 2nd stage is love, and the 3rd stage is sexual relationship and can maintain a long-lasting platonic relationship.

Lesson 2: Communication–denial Skills. This story is based on a simulation. Its objective is to develop the ability to use words to communicate one’s own feelings and thoughts appropriate to cultures and situations that includes giving comments, expressing needs, negotiating, and refusing without damaging the relationship and tendency to sex.

Lesson 3: Self-esteem Skills. This story is based on a simulation. Its objectives include improving self-esteem, unprovocative dress code for women, and refraining from going with a heterosexual friend alone and staying with a heterosexual friend in private, — all of which can lead to sexual intercourse.

Lesson 4: Self-awareness Skills. This story is based on a simulation. Its objectives are to create the ability to learn and to be aware of one’s advantages and disadvantages and what one needs or does not need. In addition, reading erotic books, watching porns, hugging, kissing, fondling, physical touching, and drinking alcoholic beverages are extremely inappropriate behaviors. If any of these happens, sexual intercourse may be inevitable.

The four lessons

The lesson plans included activity topics, learning objectives, tools/materials used, worksheets, the duration of each activity, and leaning assessments. The worksheets for each activity are as follows and copies in Thai are provided in the *Extended data* ( Neranon & Thongsong, 2021b):

1. Relationship building skills: Two worksheets were used for two activities. The first worksheet for an activity entitled ‘What stage is it?’ comprised short descriptions of relationship situations and the student needed to identify the stage that the relationship was in (i.e., Stage 1 - Friendship, Stage 2 - Love, or Stage 3 - Sexual relationship). The other worksheet was designed for an activity entitled ‘Is it appropriate?’. This activity requires the students to role play based on situations of both appropriate and inappropriate relationship building.
2. Communication–denial skills: A worksheet for an activity entitled ‘What would you say?’ was given, allowing the students to complete incomplete conversation using refusal skills for various situations.
3. Self-esteem skills: A worksheet for an activity entitled ‘I’m proud of myself’ was provided. The students needed to provide answers and reasons why they would or would not perform such behaviors given in each situation.
4. Self-awareness skills: Two worksheets were used for two activities. The first activity entitled ‘This is right!’ asked the student to identify behaviors that should or should not performed with friends of the opposite gender. The second activity entitled ‘What’s happening?’ required the students to analyze causes and provide solutions for each problem.

*Validation of CAIFLS*

Five experts in the field of preventing risky sexual behaviors in children and adolescents and educational technology were approached to validate the CAIFLS. All experts used a 39-item evaluation checklist of 5-point Likert scale developed by the researchers to assess the quality of CAIFLS in terms of content (10 items), functionality (19 items), as well as lesson plans and worksheets (10 items). It was found that the CAI content was of quality at a very good level (M = 4.44, SD = 0.206), the CAI functionality was at a very good level (M = 4.86, SD = 0.328), and the quality of the activity plans and worksheets of activities for preventing risky sexual behaviors for early adolescents was also at a very good level (M = 4.42, SD = 0.175). The researchers then improved the quality of the CAIFLS based on the suggestions given by the experts.

**Figure 1.**
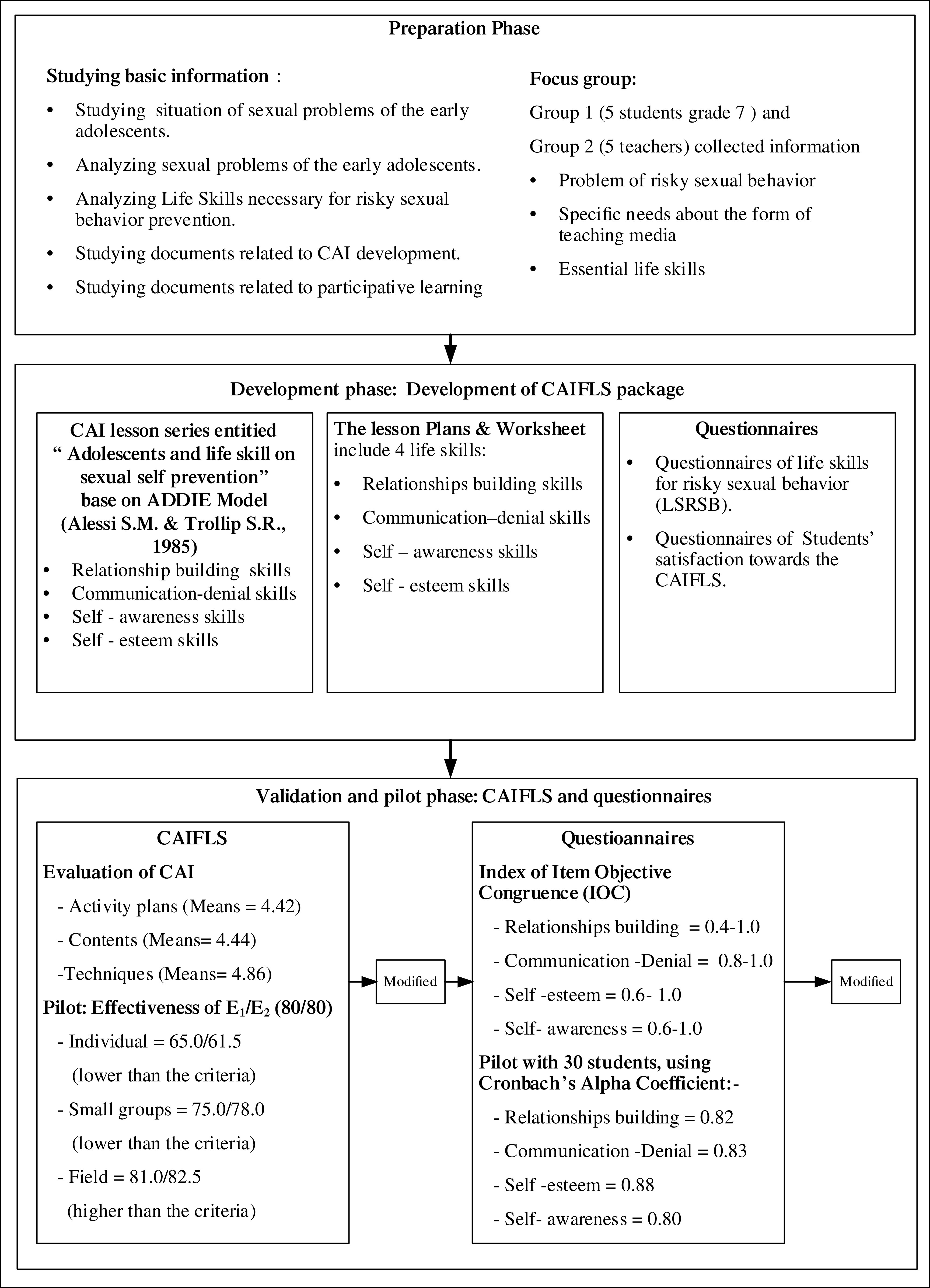
Phase 1: Development of the CAIFLS.

A pilot test of CAIFLS was performed with students in Bangkok having the same characteristics as the target participants, which were students aged 12–13 years studying in Grades 7 students in schools of Bangkok. The efficiency of the CAIFLS was examined by the students using the E1/E2 formula as follow. The findings of efficiency analysis of the CAIFLS activity package based on 80/80 criteria (or the E _1_/E _2_ formula) showed the efficiency values; the first try out with individuals (three students) gave results of 65.0/61.5, which were lower than the set criteria. Misspelled words in CAIFLS were corrected and the questionnaires were reordered. The second try out with a small group (10 students) gave results of 75.0/78.0, which were also lower than the criteria. The language used in the questions were revised for appropriateness for the students. The final try out with a larger group (30 students) gave results of 81.0/82.5, which were higher than the criteria ( Figure 1).

### Phase 2

*Participants*

Sample size was determined by the effect size of 0.55. The power analysis was set at 0.8 at the 0.05 level of significance ( Cohen, 1988) and tested with one-tailed test by using G*power to obtain 42 sample size for each group. In order to avoid losing cases, 5% of the sample (n = 3), were included into each group ( Faul et al., 2007). Therefore, the sample size was 45 participants for each group. They were assigned into the experimental and control group.

Purposive sampling of one secondary school in Bangkok was conducted. This school has been reported by teachers as having a problem with risky sex behavior. Simple random sampling was then used to select two classrooms. One classroom was the experimental group, with 44 students, and one classroom was the control group, with 43 students. The participants of the present study had a total 87 students.

*Inclusion criteria:* secondary school students aged 12–13 years studying in Grades 7 in schools of Bangkok, with the ability to read, write, and communicate.

*Development of the assessment questionnaires*

Two questionnaires were developed to analyze if the objectives of CAIFLS were successful (questionnaire 1) and to see student satisfaction with the CAIFLS (questionnaire 2) ( *Extended data* ( Neranon & Thongsong, 2021b)).

*Questionnaire 1:* A two section questionnaire. Section one contained a checklist to obtain general demographic information; section two consisted of 33 items included the following sections:

1.1 Relationship building skills on prevention for risky sexual behaviors. There were 10 items with rating scales from 1-5 (5 = Totally agree, 4 = Agree, 3 = Partly agree, 2 = Disagree, 1 = Totally disagree). The items included both positive and negative statements.
1.2 Communication–denial skills for risky sexual behavior prevention. There were 5 items of multiple choice format (1= right and 0 = wrong).
1.3 Self-esteem skills on prevention for risky sexual behaviors. There were 8 items with rating scales from 1-5 (5 = very high, 4 = high, 3 = fair, 2 = low, 1 = very low). The items included both positive and negative statements.
1.4 Self-awareness skills of risky sexual behaviors. There were 10 items with rating scales 1-5. (5 = Totally agree, 4 = Agree, 3 = Partly agree, 2 = Disagree, 1 = Totally disagree). The items included both positive and negative statements.

*Questionnaire 2:* Consisted of 10 items with rating scale 1-5 (5 = mostly satisfied, 4 = more satisfied, 3 = moderately satisfied, 2 = less satisfied, 1 = not satisfied).

*Validation of questionnaires*

Content validity of the questionnaires were evaluated by five experts in the field of preventing risky sexual behaviors in children and adolescents. All of the experts worked as child health specialists in Bangkok, including two child health and behavior specialists, two school health nurse, and one educational technology. The five experts assessed the items of the two questionnaires using the Index of Item Objective Congruence (IOC). The assessment of the questionnaire items showed that most of the items were at a high level of item-objective congruence (IOC ≥ 0.5), except only one item (IOC = 0.4) of questionnaire 1. Subsequently, the researchers revised the questionnaire items based on the experts’ feedback in terms of content, language use, and appropriate context representation for children aged 12–13 years in Bangkok.

A pilot test was performed with 30 secondary school students in Bangkok having the same characteristics as the target participants, which were students aged 12–13 years studying in Grades 7 students in schools of Bangkok, with the ability to read, write, and communicate with normal, Reliability was analyzed using Cronbach’s alpha. It was found that the reliability values of the two questionnaires were greater than 0.80, which is at a good level. The revised version of the instruments were then used for data compilation ( Figure 1).

*Data collection*

After obtaining consent from the students and their legal parents, a group of trained research assistants coordinated with the teacher coordinators of the schools. Then the researchers and research assistants went to the participants’ schools to collect data. Before starting compiling data, the researchers and research assistants gave self-introduction, expressed the purpose of the research clearly, and clarified the data collection details to each of the participants.

After having understood all the research-related information, each participant was given questionnaire 1 to complete (Pre-test) (generally 30 to complete).

The group of 44 participants assigned to the experimental group received the CAIFLS program. The participants of this group received a total of 4 lessons, 60 minutes each. The group of 43 participants assigned to the control group received no CAIFLS.

The 1st Round. The experimental group was taught through CAIFLS at the school’s computer room. The researcher explained the purpose of doing activity, duration of the activity, and learning methods according to the CAI manual. In this round, the experimental group had to do a pretest first and started learning about Lesson 1: Relationship building Skills for 20 minutes. After the lesson, the experimental group was divided into 10-11 member groups to cooperate in completing the 1st worksheet “What stage is it ?” and the 2nd worksheet “Is it appropriate?” This activity requires the students to role play base on situation of both appropriate and in appropriate relationship building in class in a 40-minute duration. Overall, this activity took 60 minutes. The researchers assessed with an observational assessment form while students role play.

The 2nd Round. The experimental group was taught through CAIFLS at the school’s computer lab. The researcher explained the purpose of doing activity and duration of the activity. Later, the experimental group learned Lesson 2: Communication–denial Skills for 20 minutes. After the lesson, the experimental group was divided into 10-11 member groups to cooperate in completing the worksheet “What would you say”. The students were also asked to discuss and make a presentation in class, allowing the students to complete or incomplete conversation using refusal skills for various situations. in a 40-minute duration. Overall, this activity took 60 minutes. The researchers assessed with read worksheet and observational form while students role play.

The 3rd Round. The experimental group was taught through CAIFLS at the school’s computer lab. The researcher explained the purpose of doing activity and duration of the activity. Later, the experimental group learned Lesson 3: Self-esteem Skills for 20 minutes. After the lesson, the experimental group was divided into 10-11 member groups to cooperate in completing the worksheet “I’m proud of myself”. The students were also asked to discuss and make a presentation in class about why they would or would not perform such behaviors given in each situation in a 40-minute duration. Overall, this activity took 60 minutes. The researchers assessed the students’ responses given in the worksheet.

The 4th Round. The experimental group was taught through CAIFLS at the school’s computer lab. The researcher explained the purpose of doing activity and duration of the activity. Later, the experimental group learned Lesson 4: Self-awareness Skills for 20 minutes. After the lesson, the experimental group was divided into 10-11 member groups to cooperate in completing the 1st worksheet “This is right!” and the 2nd worksheet “What’s happening?”. The students were also asked to discuss and make a presentation in class about causes and provide solutions for each problem in a 30-minute duration. The researchers assessed the students’ responses given in the worksheet. Afterward, the students did a posttest on CAI for 10 minutes. Overall, this activity took 60 minutes.

**Figure 2.**
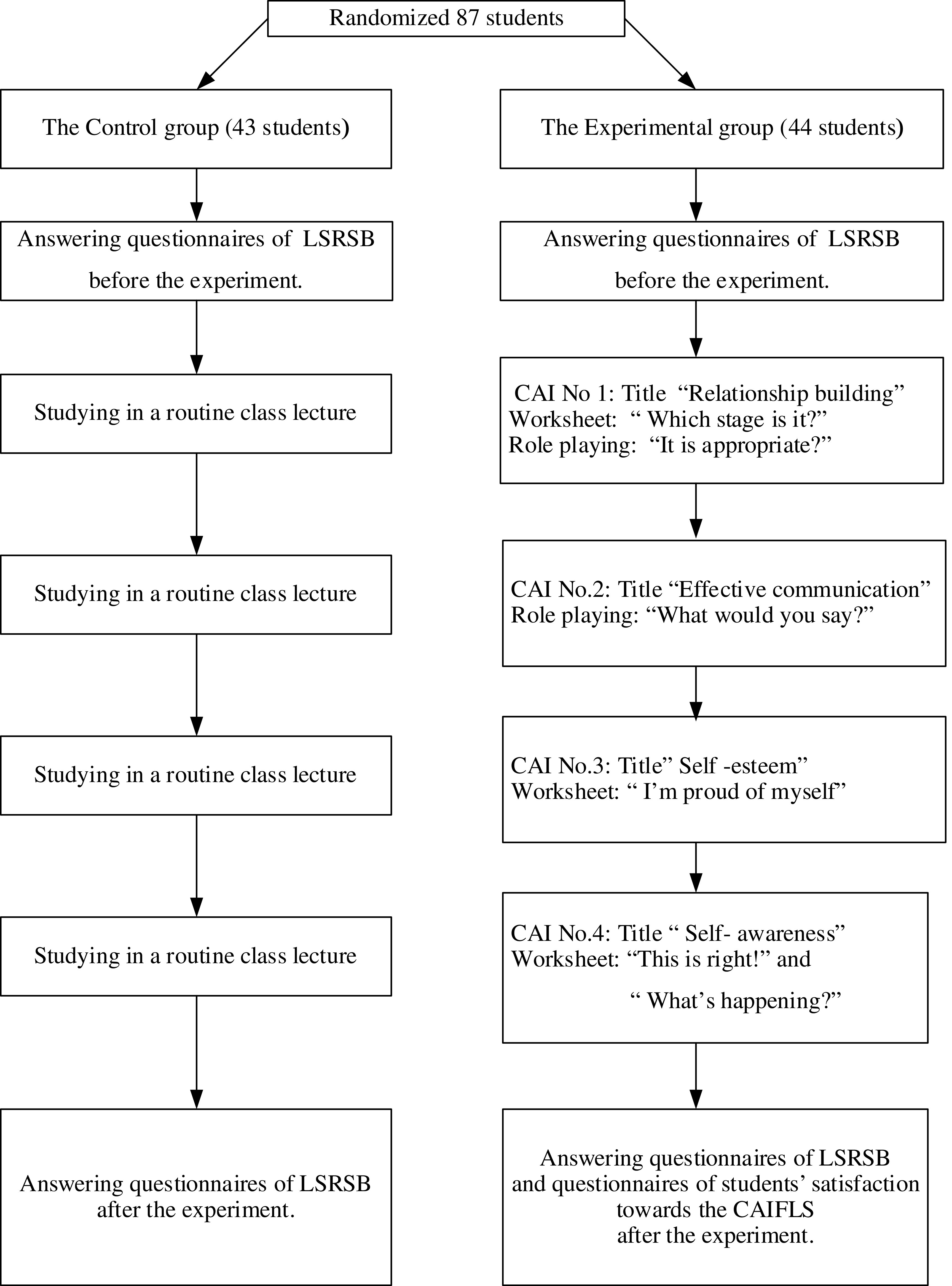
Phase 2: The CAIFLS was conducted on an experimental group.

All participants in both groups completed questionnaire 1 after the CAIFLS intervention had been completed (post-test) ( Figure 2). Students then completed questionnaire 2.

*Statistical analysis*

Descriptive statistics were utilized for data analysis of demographic information (section 1, questionnaire 1), and the level of student satisfaction on CAIFLS (questionnaire 2).

For questionnaire 1 (section 2), frequency distribution testing was done using the Kolmogorove-Smirnov Test formula, and normal curve distribution was confirmed. Therefore, a t-test at.05 level of statistically different significance was used to evaluate the effect of the CAIFLS. All statistical analyses of the present study were conducted using SPSS software version 26 ( IBM Corp., 2019).

## Results

### Demographic characteristics of the sample

The participants were 87 students who were early adolescents studying in Grade 7, in Bangkok. A total of 44 students (males 47.75%, females 52.3%) were assigned as the experimental group, and 43 students (Males 48.8%, females 51.2%) were in the control group. Their average age was 12 years. The average academic achievement (GPA) was between 2.50 and 2.99.

### Validation of CAIFLS

The efficiency (E1/E2) analysis of the CAIFLS based on 80/80 criteria with the experimental group (44 students) was 80.2/82.5, which was higher than the criteria.

### Efficacy of CAIFLS (questionnaire 1)

To test for differences before and after the intervention, mean scores of the four variables (i.e., relationship building skills, communication-denial skills, self-esteem skills and self-awareness skills) were evaluated.

Mean scores of the experimental group were as follows: for Relationship building, the pre-test was ( $\bar{X}$ = 28.16, SD = 3.12) and the post-test was ( $\bar{X}$ = 30.38, SD = 2.63), for Communication-denial, the pre-test was ( $\bar{X}$ = 2.25, SD = 0.94 ) and the post-test was ( $\bar{X}$ = 3.79, SD = 0.90), for Self-esteem, the pre-test was ( $\bar{X}$ = 22.54, SD = 2.86) and the post-test was ( $\bar{X}$ = 24.16, SD = 2.64), for Self-awareness, the pre-test was ( $\bar{X}$ = 28.16, SD = 2.79) and the post-test was ( $\bar{X}$ = 30.82, SD = 2.59), for LSRSB, the pre-test was ( $\bar{X}$ = 81.11, SD = 4.96) and the post-test was ( $\bar{X}$ = 89.16, SD = 4.06). When comparing the difference of the mean scores between the pre-test and the post-test, the mean post-test score of the experimental group was significantly higher than the mean pre-test score (p < .05). Within the control group no significant differences were found in the mean values of the four variables between pre-test and post-test scores (p > .05) ( Table 1).

**Table 1.** Mean and paired t-test statistics of the four variables of the control group and the experimental group.

Group	N	Pre-test		Post-test		t	p-value
		$\bar{X}$	S.D.	$\bar{X}$	S.D.		
**Relationship building**							
Control	43	28.90	2.75	28.93	2.82	.443	0.660
Experiment	44	28.16	3.12	30.38	2.63	5.856 **	0.001
**Communication-denial**							
Control	43	2.39	1.04	2.20	0.91	1.308	0.198
Experiment	44	2.25	0.94	3.79	0.90	9.242 **	0.001
**Self-esteem**							
Control	43	22.60	3.15	22.53	2.87	.476	0.637
Experiment	44	22.54	2.86	24.16	2.64	4.565 **	0.001
**Self-awareness**							
Control	43	28.69	3.17	29.02	2.94	-2.009	0.051
Experiment	44	28.16	2.79	30.82	2.59	6.724 **	0.001
**Total LSRSB**							
Control	43	82.60	4.44	82.69	4.02	-.321	0.750
Experiment	44	81.11	4.96	89.16	4.06	11.034 **	0.000

** p < .01.

At the end of the experiment, the mean scores between the control group and the experimental group, were compared, mean score on Relationship building of the control group was ( $\bar{X}$ = 29.21, SD = 2.95) and that of the experimental group was ( $\bar{X}$ = 30.38, SD = 2.62), mean score on Communication-denial of the control group was ( $\bar{X}$ = 2.20, SD = 0.91) and that of the experimental group was ( $\bar{X}$ = 3.79, SD = 0.90), mean score on Self-esteem of the control group was ( $\bar{X}$ = 22.53, SD = 2.87) and that of the experimental group was ( $\bar{X}$ = 24.16, SD = 2.65), mean score on Self-awareness of the control group was ( $\bar{X}$ = 29.25, SD = 2.89) and that of the experimental group was ( $\bar{X}$ = 30.82, SD = 2.59). When the mean post-test scores between two groups were compared, the post-test scores of the experimental group were higher than those of the control group with a statistical significance level (p < .05) ( Table 2).

**Table 2.** Mean and independent-samples t-test of the four variables after the intervention between the control group and the experimental group.

Group	N	$\bar{X}$	S.D.	t	p-value
**Relationship building**					
***Pre-test***				.523	.602
Control	43	28.51	3.16		
Experiment	44	28.16	3.12		
***Post-test***				-1.965*	0.043
Control	43	29.21	2.95		
Experiment	44	30.38	2.62		
**Communication-denial**					
***Pre-test***				.680	.499
Control	43	2.39	1.05		
Experiment	44	2.25	0.94		
***Post-test***				-8.135**	0.001
Control	43	2.20	0.91		
Experiment	44	3.79	0.90		
**Self-esteem**					
***Pre-test***				.092	.927
Control	43	22.60	3.14		
Experiment	44	22.54	2.86		
**Post-test**				-2.742**	0.007
Control	43	22.53	2.87		
Experiment	44	24.16	2.65		
**Self-awareness**					
***Pre-test***				.432	.667
Control	43	28.44	3.29		
Experiment	44	28.16	2.79		
***Post-test***				-2.650**	0.010
Control	43	29.25	2.89		
Experiment	44	30.82	2.59		

**p < .01, * p < .05.

### Satisfaction with the CAIFLS (questionnaire 2)

Table 3 presents the satisfaction of the experimental group with the CAIFLS package after finishing the intervention. The CAIFLS satisfaction of the experimental group was found at the level of ‘more satisfied’.

**Table 3.** CAIFLS satisfaction of the experimental group.

Items	Satisfaction score					$\bar{X}$	S.D.	Satisfaction level
	5	4	3	2	1			
1. The lesson series can engage the learners for learning.	45.50	47.70	6.80	0.00	0.00	4.39	0.62	Very high
2. The lesson series has necessary basic information, e.g. learning objectives, functions.	43.20	45.50	11.30	0.00	0.00	4.32	0.67	Very high
3. The difficulty level of the content is suitable for the target learners.	27.20	68.20	2.30	2.30	0.00	4.25	0.69	Very high
4. The content is meaningful and suitable for the target learners.	64.50	31.00	1.10	3.40	0.00	4.14	0.76	High
5. The language used in the lesson series is clear and easy to understand.	36.40	43.20	18.20	2.20	0.00	4.14	0.79	High
6. The language used in the series is suitable for the target learners.	31.80	61.40	6.80	0.00	0.00	4.25	0.58	Very high
7. The course is well-designed and well-sequenced for learning and understanding.	39.50	59.10	1.40	0.00	0.00	4.18	0.56	High
8. The instructional methods used are interesting.	22.70	70.50	6.80	0.00	0.00	4.20	0.63	High
9. The learners can review lessons by themselves freely.	20.50	59.00	20.50	0.00	0.00	4.00	0.68	High
10. The lesson series can promote learning reinforcement the learners.	13.60	77.30	9.10	0.00	0.00	4.05	0.80	High
*Total*	*34.50*	*56.30*	*8.40*	*0.80*	*0.00*	*4.20*	*0.29*	*High*

5 = mostly satisfied, 4 = more satisfied, 3 = moderately satisfied, 2 = less satisfied, 1 = not satisfied.

## Discussion

The CAIFLS lesson series developed by the researchers improved teaching efficiency in building life skills to prevent risky sexual behaviors of early adolescents. This was shown by the efficiency values from CAIFLS validation, which were above the set criteria (80.0/80.0). The experimental group had higher average post-test scores for life skills in preventing risky sexual behaviors after the intervention than the pre-test scores. The post-test scores of the experimental group were also higher than the post-test scores of the control group. This indicates that the learning process of the experiment group developed because the lesson series of CAIFLS strengthened their life skills in preventing sexually risky behaviors of early adolescents. As a newly developed and carefully designed instructional media, the CAIFLS has various tools (the CAI lesson series entitled 'Adolescents and Life Skills on Sexual Self Prevention’, group discussions, role-playing activities) to help students develop their life skills in preventing high-risk sexual behaviors.

The characteristics of CAI meet the differences between individuals, helping learners to study according to their abilities through well-arranged learning content which allows learners to learn step by step ( Kanokthavomtham & Lalognum, 2017: Krungkraipetch, 2019: Vatanasomboon P et al., 2018). Based on the results of the questionnaire on opinions and satisfaction of the experimental group towards the CAIFLS lesson series, it was found that the lesson series can engage the learners for learning. They further mentioned that they liked the colorful cartoon images along with clear voice-over. The content was divided into small lessons, making the students keep learning with interest. This is in line with Krungkraipetch (2019), who suggests that a good cartoon platform and good audio-visual score incentivizes students to learn more. The questionnaire results further showed that the lesson series had necessary basic information, e.g. learning objectives, functions. The CAIFLS lesson series had clear instructions, learning objectives, main menu, submenu, and exit buttons, which is user-friendly for self-learning without time restrictions for learning. This is supported by previous research ( Sukpom T et al., 2017: Wongjunthong K & Rachawijit J.,2010) that studied the effects of learning achievement on sex education in Grade 8 students through a computer-assisted instruction program, which revealed that the students enjoyed learning through the CAI learning program because its instructions and learning content were easy to understand. Another prominent feature of the CAIFLS is that the difficulty level of the content and the language used in the series was suitable for the target learners. The participants of the experimental group agreed that the lessons were easy to follow and understand, and topics of the lessons were related to their everyday life. In addition, based on our observation, the majority of students paid attention to the lessons while learning. They did not talk to their peers but enjoyed the lessons as they laughed or smiled. However, the participants of the study were quite worried about taking the tests after lessons. Some took longer time to complete the tests as they were afraid of giving wrong answers.

With regard to relationship building skills, it was found that the experimental group had higher post-test scores than their pre-test, and they also had significantly higher post-test scores than the control group. This can be because simulations were assigned through the CAI topic entitled *Relationships building.* The content was a scenario to learn how to build good relationships with each other in order to understand the differences between three stages of relationships (i.e., Stage 1 - Friendship, Stage 2 - Love, and Stage 3 - Sexual relationship) and to maintain friendships or non-sexual relationships. After completing the lesson, the participants of the experimental group were divided into small groups to discuss the situation given in the activity entitled “Which state is it?” and compose dialogs based on their discussion. Subsequently, each group performed their role-play for the activity entitled “Is it appropriate?” using their own dialogs in front of the class. This activity indicated that the practice in this aspect was relevant to real-life situations ( Chiengta et al., 2018). The students were able to exchange their competencies to solve problems and to select appropriate approaches in building better relationships with other people without breaking them ( Mahanta et al., 2016; Kosalavit et al., 2017; Sirited, 2019). The researchers observed the students as they were role-playing and found that the students seemed to have a good tendency to express friendships with their peers. However, there were some groups of students who expressed their adolescent emotions, fieriness, and also needs for peer acceptance, expressing themselves for a loving relationship.

For communication–denial skills, the experimental group had higher post-test scores than their pre-test, and they also had significantly higher post-test scores than the control group. The simulations were assigned under the CAI topic *Effective Communication.* The content allowed the students to appropriately communicate their thoughts to the situations given, using negotiation or denial skills. After the CAI lesson, small group discussions were gathered to discuss problems and to compose dialogues given in the worksheets ( *Think Hard*), and to practice a communication skill of *Saying No* (or Denial) which exists in everyday life. The students in each group performed role plays based on their own dialogues in front of the class. Consequently, they had opportunities to exchange opinions focusing on the actual practices ( Kosalavit et al., 2017; Heednakham et al., 2015), which made them build their own knowledge ( Sasinun et al, 2013). This is in line with Bualoy *et al*. (2014) who investigated the effectiveness of a sex education program to prevent sexual risk behavior on Grade 8 students in Sampran District, Nakhonpathom Province. They used real sexual behavior problems with the students and found that the students had risky sexual behaviors at a lower rate. The present study showed the same results in that most students had a good tendency to express their negotiations or denials, but there were still some students that used words to communicate in an aggressive way, for instance some female students pushed male student's hands and aggressively blamed them immediately, showing inappropriate communication skills in real situations.

As regards to self-esteem skills, the findings showed that the experimental group had significantly higher post-test scores than their pre-test, and they also had significantly higher post-test scores than the control group. Simulations were assigned under the CAI topic *Self-esteem.* The lesson was to make the students aware of self-value, for example, not to dress provocatively or hang out with a friend of opposite gender alone or in a private place. The students of the experimental groups developed the self-esteem skill in small groups following the instructions given in the worksheet. The students were required to think critically, share their opinions, and expressed their thoughts on the flipcharts to confirm their thoughts and self-values relating to non-conformities to risky sexual behaviors ( Olanratmanee, 2018: Thamrongsotthisakul W., 2017). The researchers examined the students' responses given in the worksheet of the lesson and found that most students had good prospects. They were able to provide reasons for self-esteem that they would not behave inappropriately. For example, a female student said that she would not dress provocatively due to an increase in number of rape and sexual assault cases on girls.

For self-awareness skills, the experimental group had significantly higher post-test scores than their pre-test, and they also had significantly higher post-test scores than the control group. The simulations were assigned through the CAI topic entitled *Self-awareness.* The lesson made the students aware of their strengths and weaknesses as well as their needs and unwanted needs. The lesson also let the students aware that watching sex videos, hugging, kissing, touching body parts, or drinking alcoholic beverages were examples of inappropriate sexual behaviors which can lead to sexual problems, e.g. sexual intercourse or school-age pregnancy. After the CAI lesson, the students discussed problems provided in the worksheet in small groups. During the activity, the students brainstormed many ideas about risky sexual behaviors in various situations. Through this activity, the students analyzed their own weaknesses and strengths. They comprehended more clearly about their own needs and individual differences of other people ( Bualoy et al., 2014: Phumjuntuk & Duangsong R, 2011: Widman L et al, 2016). The students in each discussion group shared their constructive ideas to find appropriate approaches to protect themselves from risky sexual behaviors. The researchers examined the student's responses in the worksheet of the lesson and discovered that students had a good tendency to identify appropriate and inappropriate behaviors. They were also able to tell what to do in order not to put themselves in a situation where there is a risk of sex.

Based on the findings of the present study, one conclusion can be made that the CAIFLS package can be used as an effective tool to prevent risky sexual behaviors in early adolescents. However, it can also be concluded that CAIFLS package should not be used as a single learning source since it might limit learning or correct understanding of the students towards the topics. It needs to be used by a knowledgeable teacher since it requires human interactions, discussions, question and answer sessions, and role-playing activities involved in order to effectively promote life skills for risky sexual behavior prevention in Thai early adolescents for their better understanding.

### Recommendations for future research

This study evaluated the effectiveness of the CAIFLS learning package with Grade 7 students in only one school in a Bangkok district. This CAIFLS learning package should be further used in other districts and in more schools.

Although the CAIFLS learning package can be adopted to promote life skills for risky sexual behavior prevention, teachers that intend to use this learning package are encouraged to be trained how to use this learning package effectively before use in their classrooms.

## Conclusions

It can be confirmed that the CAIFLS can be effectively applied as an educational media for teachers, pediatric nurses, school health nurses, and related school personnel to be used for promoting life skills of the early adolescents to prevent risky sexual behaviors in schools or communities.

Limitation of the most contents of CAIFLS were developed from the adults point of views based on Thai cultural norm saying that sexual intercourse among children and risky sexual behaviors of early adolescents are inappropriate and early adolescents must refrain from risky sexual behaviors at all.

## Data availability

### Underlying data

Underlying data cannot be shared as the ethical committee that approved this study states that only aggregated data can be shared openly. In addition, the consent form that guardians/children signed explicitly stated that the data resulting from the study would not be openly shared. Researchers interested in accessing the data will need to submit an official letter of request for the data to Navamindradhiraj University, and will be asked to confirm that they will not violate the ethical standards of the ethical committee and protect the anonymity of the participants. Researchers can contact the corresponding author, who can facilitate this process.

### Extended data

Open Science Framework: Development of activity package computer assisted instruction for life skills to prevent risky sexual behaviors on early adolescents, Bangkok, Thailand, https://doi.org/10.17605/OSF.IO/24XPY ( Neranon & Thongsong, 2021b).

This project contains the following extended data:

- Interview questions for Thai students and teachers (in Thai)
- Questionnaires CAIFLS for Early Adolescents, research instrument in English
- Worksheets for CAIFLS (in Thai)
- Lesson plan for Thai early adolescent (Thai version)

Data are available under the terms of the Creative Commons Zero “No rights reserved” data waiver (CC0 1.0 Public domain dedication).

## Software availability

CAIFLS program available from: https://docs.google.com/forms/d/16NLKoSJjW49QSULlSkOUFPXnVDvmUuddaHXnCy9WHac/edit

Source code available from: https://github.com/Neranon/WN-CAIFLS

Archived source code as at time of publication: http://doi.org/10.5281/zenodo.4539903 ( Neranon & Thongsong, 2021a).

License: MIT
