# Supplementary material for: Development of a computer-assisted instructional package for life skills to prevent risky sexual behaviors in early adolescents, Bangkok, Thailand
Source: F1000Res. 2022 Jan 11;10:385. Originally published 2021 May 13. [Version 2] doi: 10.12688/f1000research.27773.2 (PMC8767427; doi:10.12688/f1000research.27773.2)
Supplement: Peer review history

# Peer review: Reviewer response for version 2

**DOI:** 10.5256/f1000research.119835.r119404

**Published:** 2022-01-18

**Authors:** Saowaluck Settheekul

**Affiliations:** 1 Faculty of Nursing, Chiang Mai University, Chiang Mai, Thailand

The authors revised this article more clearly. I have no further comment on this version.

Is the work clearly and accurately presented and does it cite the current literature?

Partly

If applicable, is the statistical analysis and its interpretation appropriate?

Yes

Are all the source data underlying the results available to ensure full reproducibility?

Yes

Is the study design appropriate and is the work technically sound?

Yes

Are the conclusions drawn adequately supported by the results?

Partly

Are sufficient details of methods and analysis provided to allow replication by others?

Partly

Reviewer Expertise:

Health promotion and sexual health

I confirm that I have read this submission and believe that I have an appropriate level of expertise to confirm that it is of an acceptable scientific standard.

# Peer review: Reviewer response for version 1

**DOI:** 10.5256/f1000research.30712.r95357

**Published:** 2021-09-28

**Authors:** Christopher Fuster Bueno

**Affiliations:** 1 University of Northern Philippines, Vigan, Philippines

**Development of a computer-assisted instructional package for life skills to prevent risky sexual behaviors in early adolescents, Bangkok, Thailand**

 The study provides a clear and accurate presentation on cited current literature along with the lesson intervention on the computer-assisted instructional package to prevent risky sexual behaviors with positive findings on previous researches. The research design introduces the CAILFS Model to propose and analyze the complementation of the criterion difference on academic achievement and life skills with the use of computer-assisted lesson activities in preventing sexually risky behaviors. It has sufficient details to interpret the factual triangulation of information that can be replicated utilizing the 21st-century teaching-learning strategies on the computer-assisted instructional package with deliberate responsive life skills to prevent risky sexual behaviors.

 The research replication of the utilization of a computer-assisted instructional package will also enhance the Bloom Taxonomic Model on Higher Order Thinking Skills (HOTS) on effective domain based on the discussion of the life skills. Furthermore, psychomotor and cognitive domains are highly reflective of the computer-assisted instructional packages. It has utilized the descriptive analysis with triangulated analysis on student satisfaction on CAIFLS and used the Kolmogorove-Smirnov Test formula with interesting results on the academic interpretation about the CAIFLS efficacy to prevent risky sexual behaviors. The source of data ensures full reproducibility and the conclusion drawn provides concise results which confirmed the effectiveness of the CAIFLS model applied to the nursing academic community in promoting life skills to prevent risky sexual behaviors.

Is the work clearly and accurately presented and does it cite the current literature?

Yes

If applicable, is the statistical analysis and its interpretation appropriate?

Yes

Are all the source data underlying the results available to ensure full reproducibility?

Yes

Is the study design appropriate and is the work technically sound?

Yes

Are the conclusions drawn adequately supported by the results?

Yes

Are sufficient details of methods and analysis provided to allow replication by others?

Yes

Reviewer Expertise:

Social Science, Education and Public Administration

I confirm that I have read this submission and believe that I have an appropriate level of expertise to confirm that it is of an acceptable scientific standard.

# Response

**Published:** 2021-10-04

**Authors:** Ladaporn Thongsong

**Affiliations:** Department of Pediatric Nursing, Kuakarun Faculty of Nursing, Navamindradhiraj University, Thailand

Dear Reviewer

 I am grateful for your guidance. Your guidance is very helpful in our study. 

 Best regards.

# Peer review: Reviewer response for version 1

**DOI:** 10.5256/f1000research.30712.r86597

**Published:** 2021-06-21

**Authors:** Saowaluck Settheekul

**Affiliations:** 1 Faculty of Nursing, Chiang Mai University, Chiang Mai, Thailand

This research and development study presents how to develop and evaluate CAIFLS among adolescents. Overall, the presentation is well structured, and the discussion part is well balanced and covers the life skills aspects. A few more points may be discussed:

1. The introduction started talking about adolescent pregnancy rates and its consequences and continued with the importance of life skills to prevent risky sexual behaviours. So, there needs to be more explanation about adolescent sexual risk behaviours in Bangkok and Thai cultural norms related to sexual risk behaviours.
2. Methods - Authors should explain more about the contents and activities of each CAIFLS's lessons, especially the content related to risky sexual behaviours and how role-playing was performed, and how researchers observed the participants.
3. Results - Authors should rewrite results from t-test analysis based on one-tailed hypothesis tests.
4. The limitation of Thai cultural norms should be presented because most contents of CAIFLS were developed based on adults' s perspectives that pre-coital or sexual risk behaviours among adolescents are shamed and prohibited.

Is the work clearly and accurately presented and does it cite the current literature?

Partly

If applicable, is the statistical analysis and its interpretation appropriate?

Yes

Are all the source data underlying the results available to ensure full reproducibility?

Yes

Is the study design appropriate and is the work technically sound?

Yes

Are the conclusions drawn adequately supported by the results?

Partly

Are sufficient details of methods and analysis provided to allow replication by others?

Partly

Reviewer Expertise:

Health promotion and sexual health

I confirm that I have read this submission and believe that I have an appropriate level of expertise to confirm that it is of an acceptable scientific standard, however I have significant reservations, as outlined above.

# Response

**Published:** 2021-07-01

**Authors:** Ladaporn Thongsong

**Affiliations:** Department of Pediatric Nursing, Kuakarun Faculty of Nursing, Navamindradhiraj University, Thailand

Dear Reviewer

 I am grateful for your guidance. Your guidance is very helpful in our study. We will be happy to modify the work according to your suggestions.

 Best regards.

# Response

**Published:** 2021-12-27

**Authors:** Ladaporn Thongsong

**Affiliations:** Department of Pediatric Nursing, Kuakarun Faculty of Nursing, Navamindradhiraj University, Thailand

The revised part as suggested by reviewer 1

1. We have revised the paper based on the suggestions from reviewer 1.

The revised part as suggested by reviewer 2

1. The introduction: explains more about the risky behavior of adolescents in Bangkok, and explain Thai cultural norms about risky sexual behavior.
2. The methods: explains more about the contents in topic CAI lesson series entitled Adolescents and life skills on sexual self-prevention, and activities of each CAIFLS’s lessons in topic Data collection. Added lesson plans in Extended data and updated DOI in the new version.
3. Results: I rewrote results from t-test analysis base on hypothesis test.
4. Conclusions: I added the limitation of Thai cultural norms accordingly reviewer's suggestion.
